# Nie wieder postoperative Radiotherapie in der pN2-Situation nach kompletter Resektion des nichtkleinzelligen Bronchialkarzinoms? Eine strahlentherapeutische Interpretation des Lung ART Trial

**DOI:** 10.1007/s00066-022-01924-3

**Published:** 2022-04-01

**Authors:** Fabian Weykamp, Sebastian Adeberg

**Affiliations:** Klinik für Radioonkologie und Strahlentherapie, Universitätsklinikum Heidelberg, Im Neuenheimer Feld 400, 69120 Heidelberg, Deutschland

## Hintergrund und Fragestellung

Trotz perioperativer Chemotherapie kommt es bei einem Großteil der Patienten mit einem NSCLC im Stadium III nach Resektion zu einem Rezidiv. Die Datenlage zum Nutzen einer zusätzlichen postoperativen Radiotherapie (PORT) kurz vor der Jahrtausendwende stellte sich widersprüchlich dar: Während die PORT im pN2-Stadium das Lokalrezidivrisiko senkte, verminderte sie im pN0–1-Stadium hingegen das Gesamtüberleben [1]. Die CALGB-9734-Studie sollte hier mehr Klarheit bringen, wurde jedoch aufgrund von Rekrutierungsproblemen frühzeitig geschlossen. In einer Subgruppenanalyse der ANITA-Studie konnte zuletzt 2008 ein „benefit“ für das Gesamtüberleben im pN2-Stadium oder im pN1-Stadium bei ausgebliebener Chemotherapie nachgewiesen werden [2].

## Patientengut und Methoden

Diese prospektive, multizentrische und randomisierte Phase-III-Studie rekrutierte von 2007–2018 Patienten aus 64 europäischen Kliniken. Eingeschlossen wurden Patienten mit kompletter Resektion ihres NSCLC, aber Nachweis eines pN2-Status. Der primäre Endpunkt der Studie war eine Überlegenheit des „disease-free survival“ (DFS) im Bestrahlungsarm, in welchem normofraktioniert 54 Gy auf das Mediastinum appliziert wurden.

## Ergebnisse

501 Patienten wurden in die Studie eingeschlossen. Das mediane Follow-up betrug 4,8 Jahre. Das primäre Studienziel wurde verfehlt. Im PORT-Arm betrug das DFS nach 3 Jahren 47 % und im Kontrollarm 44 % (Unterschied nicht signifikant).

## Schlussfolgerung der Autoren

Eine zusätzliche konformale, postoperative Radiotherapie (PORT) kann nach R0-Resektion eines NSCLC im Stadium IIIA N2 nicht als Therapiestandard bezeichnet werden.

## Kommentar

Die Pressemitteilung zum Abstract der vorliegenden Studie vom ESMO 2020 lautete: „No benefit for post-operative radiotherapy in non-small-cell lung cancer“. Ist dies auch die zentrale Botschaft, die wir uns heute aus der Vollpublikation ableiten sollten? Denn die PORT beim pN2-Status nach kompletter Resektion eines NSCLC bleibt eine komplexe Thematik. Auch die aktuellste „NCCN guideline“ äußert sich bislang zurückhaltend und verweist auf einen anhaltenden Review-Prozess. Die Datenlage hierzu stellte sich über die letzten zwei Jahrzehnte dürftig da.

Zuletzt konnte man sich bezüglich der PORT auf eine retrospektive strahlentherapeutische Auswertung der ANITA-Studie berufen. Diese erlaubte die Argumentation, dass eine PORT in der pN2-Situation durchzuführen sei oder beim pN1-Status, falls keine perioperative Chemotherapie erfolgen konnte. Etwa zur gleichen Zeit wie die Lung-ART-Studie wurde zur gleichen Thematik auch die PORT-C-Studie aus China publiziert, mit einem ähnlichen Resultat [3]. Hierbei muss jedoch beachtet werden, dass beide Studienpopulationen hoch selektiert waren. In der PORT-C-Studie war es sogar Voraussetzung, dass die Patienten vor der PORT die volle adjuvante Chemotherapie (4 Zyklen) erhalten hatten, was wohl kaum die klinische Behandlungsrealität widerspiegeln dürfte.

Zudem findet beim NSCLC, anders als bei vielen anderen Tumorerkrankungen, die Zahl der befallenen Lymphknoten keine Berücksichtigung bei der Beschreibung des Nodalstatus. Oftmals behilft man sich daher mit dem Begriff des „Multi-Level-Befalls“, um ein hohes Rezidivrisiko anzuzeigen. In einer retrospektiven Studie aus Japan konnte gezeigt werden, dass beim Multi-Level-Befall das 5‑Jahres-DFS ohne PORT 6 % statt 41 % nach erfolgter PORT beträgt [4]. Genauso, wie beide Studien gezeigt haben, dass eine pauschale PORT in der pN2-Situation nicht ratsam ist, kann somit aber auch nicht von einem pauschalen pN2-Status gesprochen werden.

Was wir nun allerdings angesichts beider Studien auch für unsere heutige Zeit wissen, ist, dass die Lokalrezidivrate durch die PORT halbiert wird, also lokal wirksam ist. Gleichzeitig weist der direkte Vergleich beider Studien darauf hin, dass die IMRT, welche zu 90 % in der PORT-C-Studie angewandt wurde, eine deutlich geringere Toxizität aufweist als die 3‑D-konformale Bestrahlung (89 % in der Lung-ART-Studie).

## Fazit

Die Lung-ART-Studie sollte nicht dazu genutzt werden, Patienten nach kompletter Resektion eines NSCLC N2 die PORT pauschal zu verwehren. Nach wie vor muss im interdisziplinären Tumorboard geklärt werden, ob im Einzelfall eine PORT doch sinnvoll sein kann. Die Lung-ART-Studie sollte vielmehr im Kontext mit der PORT-C-Studie genutzt werden, um für besondere Fälle den Vorzug einer IMRT zu begründen.

*Fabian Weykamp und Sebastian Adeberg, Heidelberg*
